# Renal miR-148b is associated with megalin down-regulation in IgA nephropathy

**DOI:** 10.1042/BSR20181578

**Published:** 2018-11-14

**Authors:** Lu Wen, Zhanzheng Zhao, Jing Xiao, Zheng Wang, Xiangfei He, Henrik Birn

**Affiliations:** 1 Department of Nephrology, The First Affiliated Hospital of Zhengzhou University, Zhengzhou, China; 2 Department of Biomedicine, Aarhus University, Aarhus, Denmark; 3 Department of Urology, The First Affiliated Hospital of Zhengzhou University, Zhengzhou, China; 4 Department of Renal Medicine, Aarhus University Hospital, Aarhus, Denmark

**Keywords:** cell transfection, IgA nephropathy, megalin, microRNA, renal proximal tubule

## Abstract

Megalin is essential for proximal tubule reabsorption of filtered proteins, hormones, and vitamins, and its dysfunction has been reported in IgA nephropathy (IgAN). miR-148b has been shown to regulate renal megalin expression *in vitro* and in animal models of kidney disease. We examined a potential role of miR-148b and other miRNAs in regulating megalin expression in IgAN by analyzing the association between megalin and miR-148b, miR-21, miR-146a, and miR-192 expression. Quantitative PCR (qPCR) analysis identified a marked increase in renal levels of several miRNAs, including miR-148b, miR-21, miR-146a, and a significant decrease in *megalin* mRNA levels in IgAN patients when compared with normal controls. By multiple linear regression analysis, however, only renal miR-148b was independently associated with megalin mRNA levels in IgAN. Proximal tubule megalin expression was further evaluated by immunofluorescence labeling of biopsies from the patients. The megalin expression was significantly lower in patients with highest levels of renal miR-148b compared with patients with lowest levels. To examine the direct effects of the miRNAs on megalin and other membrane proteins expression, proximal tubule LLC-PK1 cells were transfected with miR-148b, miR-21, miR-146a, or miR-192 mimics. Transfection with miR-148b mimic, but not the other three miRNA mimics inhibited endogenous megalin mRNA expression. No significant effect of any of the four miRNA mimics was observed on cubilin or aquaporin 1 (AQP1) mRNA expression. The findings suggest that miR-148b negatively regulates megalin expression in IgAN, which may affect renal uptake and metabolism of essential substances.

## Introduction

IgA nephropathy (IgAN) is the most common form of proliferative glomerulopathy worldwide, particularly in Asian populations [1], with 15–40% of patients progressing to end-stage renal disease (ESRD) within 10–20 years [2]. Proteinuria is the most important prognostic factor in IgAN [3,4]. There is evidence that the proximal tubule is a direct target of injury in IgAN [5]. Hence, proteinuria in IgAN may reflect both glomerular damage as well as proximal tubule injury leading to increased glomerular filtration and decreased tubular reabsorption, respectively.

Proximal tubule uptake of almost all filtered proteins is mediated by the multiligand endocytic receptor megalin and its co-receptor cubilin [6]. Megalin is a large, transmembrane endocytic receptor, highly expressed in the apical membranes of proximal tubule cells and important for the tubular recovery of filtered proteins, hormones, and vitamins [6–8]. Impaired megalin function has been reported in a number of acquired and common kidney disorders [6,9,10]. A previous study has suggested increased urinary excretion of megalin in IgAN associated with glomerular mesangial hypercellularity [11]. However, to our knowledge no studies have examined in detail the proximal tubule megalin expression in IgAN.

MicroRNAs (miRNAs) are endogenous, small (20–22 nts) noncoding RNAs that modulate gene expression at the post-transcriptional level through degradation or by repressing translation of target mRNAs [12]. Accumulating evidence indicates that miRNAs are directly involved in the pathogenesis, e.g. renal fibrosis, and development of many kidney diseases [13–16]. However, few studies have assessed the effects of miRNAs on megalin expression in kidney diseases. We previously identified the *megalin* gene as a target of miR-148b and showed that transfection of renal proximal tubule cells with miR-148b *in vitro* caused a down-regulation of *megalin* mRNA and protein expression, indicating that miR-148b may be involved in the regulation of proximal tubule protein reabsorption in renal disease conditions with increased levels of miR-148b [17].

Significant increases in miR-21, miR-146a, and miR-192 have been reported in kidney tissue from patients with IgAN [16,18,19]. These three miRNAs are implicated in driving renal fibrosis through profibrotic signaling pathways [16,20,21]. Based on previous findings, the present study explores the associations between renal megalin expression and miR-148b, miR-21, miR-146a, and miR-192 levels in IgAN patients. We further evaluated this association by examining the effects of the four miRNAs on megalin expression in proximal tubule LLC-PK1 cells transfected with the four miRNA mimics.

## Materials and methods

### Patients and samples

The study was approved by the ethics committee of the First Affiliated Hospital of Zhengzhou University, China and was conducted in accordance with the Declaration of Helsinki. Written informed consent was obtained from all participants prior to sample collection. This cross-sectional study included 70 patients with IgAN confirmed by kidney biopsy, from which an additional biopsy was available for research, at the First Affiliated Hospital of Zhengzhou University between December 2014 and November 2017. Patients with other coexisting renal pathology or recurrent IgAN after kidney transplantation were excluded. Clinical data including age, gender, mean arterial pressure (MAP), serum creatinine level, and 24-h urinary protein were recorded at the time of kidney biopsy. The estimated glomerular filtration rate (eGFR) was calculated using the CKD-EPI formula [22]. Kidney tissue specimens were collected from the all patients along with the clinical, kidney biopsy. Whole-stream, early-morning urine specimens for assessment of miR-148b levels were collected on the morning of kidney biopsy. Normal kidney tissue from nephrectomy specimens of 20 patients with renal cell carcinoma served as biopsy controls and urine samples from 23 healthy sex- and age-matched volunteers were included as healthy controls. Both biopsy controls and healthy controls were enrolled in the same time period as the IgAN patients. The kidney tissue and urine specimens were immediately frozen at −80°C until further analysis.

### Cell transfection

Based on the conserved seed match of miR-148b at the megalin-3′-UTR in humans and pigs, we performed cell transfection using the LLC-PK1 cell line provided by Dr J. Øivind Moskaug (University of Oslo, Oslo, Norway) [17,23]. The LLC-PK1 cells originate from the porcine kidney proximal tubule, expressing endocytic active megalin [23]. The culture and incubation of the cells were performed as previously described [17]. LLC-PK1 cells were seeded in T25 flask 24 h before transfection with miRNA mimic or its negative control (Life Technologies, Carlsbad, CA) as previously described [17]. To obtain similar cellular miRNA levels, cells were transfected with miR-148b, miR-21, miR-146a, miR-192 mimic or respective negative control at a final concentration of 10, 7, 3, and 5 nM, respectively. These transfection concentrations were based on preliminary experiments determining the cellular miRNA levels using various concentrations. A blank control was included as a reference. Cells were harvested 48 h post-transfection and used for total RNA extraction. Each experiment was performed at least three times with three to six replicates per group.

### Total RNA extraction and quantitative PCR analysis

Quantitative PCR (qPCR) experiments in IgAN patients, biopsy and healthy controls were performed by KangChen Bio-tech (Shanghai, China). Briefly, total RNA was purified from kidney tissue using TRIzol® Reagent (Thermo Fisher Scientific, Waltham, MA) for quantitation of miR-148b, miR-21, miR-146a, miR-192 and *megalin* mRNA. Urine samples were centrifuged at 12000 ***g*** for 10 min at 4°C and the sediment was discarded. TRIzol® LS Reagent (Thermo Fisher Scientific) was used for the extraction of total RNA from urine supernatant for quantitation of miR-148b. For miRNA, reverse transcription was performed with 20 µl reaction including 300 ng total RNA and 0.3 µl (1 µM) specific reverse transcription primers. For mRNA, 20 µl reaction including 1.5 µg total RNA and 1 µl (50 µM) Oligo(dT)_18_ primer or random hexamer primer (Thermo Fisher Scientific) was used for reverse transcription. qPCR was performed using the ViiA 7 Real-Time PCR System and QuantStudio™ 5 Real-Time PCR System (Applied Biosystems, Foster, CA) for miRNA and mRNA, respectively. The qPCR experiments in LLC-PK1 cells were conducted as previously described [17]. Specifically, TaqMan microRNA Assay (Applied Biosystems) was used to measure miR-148b, miR-21, miR-146a, and miR-192 levels in cells transfected with corresponding miRNA mimics and respective negative control. Meanwhile, megalin, cubilin and aquaporin 1 (*AQP1*) mRNA levels were also measured in these transfected cells. Pilot studies evaluated renal and urinary levels of several RNAs for normalization, including U6 snRNA, RNU48, and miR-16, and showed that the U6 snRNA levels varied the least across all samples (data not shown). Thus, the levels of the four miRNAs in kidney tissue, urine, and cells were normalized to U6 snRNA. The levels of *megalin* mRNA in kidney tissue were normalized to 18S rRNA, while the levels of megalin, cubilin, and *AQP1* mRNA in LLC-PK1 cells were normalized to GAPDH. The relative changes in miRNA and mRNA were calculated by 2^−ΔΔ*C*t^, where Δ*C*t = *C*t _miRNA/megalin/cubilin/AQP1_ –*C*t _U6/18S/GAPDH_ and ΔΔ*C*t = Δ*C*t _experimental_ – Δ*C*t_ control_. Primers were purchased from KangChen Bio-tech or from Eurofins Genomics (Ebersberg, Germany); the sequence was shown in Table 1.

**Table 1:** Primers used for reverse transcription and qPCR

Primer	Sequence 5′–3′
**Reverse transcription with kidney tissue and urine samples**	
miR-148b	GTCGTATCCAGTGCGTGTCGTGGAGTCGGCAATTGCACTGGATACGACACAAAG
miR-21	GTCGTATCCAGTGCGTGTCGTGGAGTCGGCAATTGCACTGGATACGACTCAACA
miR-146a	GTCGTATCCAGTGCGTGTCGTGGAGTCGGCAATTGCACTGGATACGACAACCCAT
miR-192	GTCGTATCCAGTGCGTGTCGTGGAGTCGGCAATTGCACTGGATACGACGGCTGTC
U6	CGCTTCACGAATTTGCGTGTCAT

Primer	Sense (sequence 5′–3′)	Antisense (sequence 5′–3′)
**qPCR with kidney tissue and urine samples**		
miR-148b	GGGTCAGTGCATCACAGAA	CAGTGCGTGTCGTGGAG
miR-21	GGGGGGTAGCTTATCAGACTG	CAGTGCGTGTCGTGGAGT
miR-146a	GGGTGAGAACTGAATTCC	TGCGTGTCGTGGAGTC
miR-192	GGGGCTGACCTATGAATTG	CAGTGCGTGTCGTGGAGT
U6	GCTTCGGCAGCACATATACTAAAAT	CGCTTCACGAATTTGCGTGTCAT
Megalin	AACGAGACGCACAGTAGTTCAG	AGTAGTGTTTATTACAGGCAAGAGG
18S	CAGCCACCCGAGATTGAGCA	TAGTAGCGACGGGCGGTGTG
**qPCR with cells**		
Megalin	CTGCTCTTGTAGACCTGGGTTC	TCGGCACAGGTACACTCATAAC
Cubilin	AGGAGCAACACTGAGTGTAC	AAGGGCCAGACTGAAATCG
AQP1	TTGGGCTGAGCATTGCCACGC	CAGCGAGTTCAGGCCAAGGGAGTT
GAPDH	GGCATGGCCTTCCGTGTCCCC	CACCCTGTTGCTGTAGCCAAATTC

### Immunofluorescence

Megalin protein expression in the kidney was evaluated by immunofluorescence on selected renal biopsy specimens. Patients were ranked based on renal levels of miR-148b from the lowest to the highest. Kidney biopsy sections from the ten patients with the lowest and the ten patients with the highest renal miR-148b expression, respectively, were selected for analysis. The paraffin-embedded sections (2 µm) were deparaffinized, rehydrated, and stained for megalin as previously described [24]. Sections were incubated with primary sheep anti-rat megalin antibody [17] followed by Alexa Fluor–conjugated donkey anti-sheep IgG (Life Technologies) and fluorescein-labeled lotus tetragonolobus lectin (LTL; Vector Laboratories, Burlingame, CA). Immunofluorescent images were acquired using Leica TCS SP2 confocal microcopy system (Leica Microsystems, Wetzlar, Germany) and quantitated by ImageJ 1.47 Software (National Institutes of Health, Bethesda, MD). The intensity of the megalin staining in each section was normalized to the LTL staining representing the number of proximal tubule profiles in the section.

### Statistical analyses

Data were analyzed using the SPSS v.19.0 software (IBM Corp., Armonk, NY). Data are presented as mean ± S.D. or median (interquartile range). Renal *megalin* mRNA levels were skewed and thus logarithmically transformed to conform more closely to a normal distribution. Chi-square test was used to compare categorical variables. For normally distributed data, *t*test or ANOVA was used for comparison between groups. The Mann–Whitney U-test and Kruskal–Wallis test were used for data with non-normal distribution. The Bonferroni method was applied to correct for multiple comparisons. Spearman’s coefficient was employed to analyze correlation amongst various parameters. The associations between renal *megalin* mRNA levels (dependent variable) and possible predictors, including miR-148b, miR-21, miR-146a, and miR-192 (independent variables), were analyzed by multiple linear regression analysis. The enter method was used to build the model and both the dependent and independent variables were logarithmically transformed to conform to the requirements of the model. A *P*-value less than 0.05 was considered statistically significant.

## Results

### Demographic and clinical data of the study subjects

Demographic and clinical data of the healthy controls, biopsy controls, and IgAN patients at baseline are summarized in Table 2. MAP, serum creatinine as well as 24-h urinary protein levels were higher, and eGFR was lower in both IgAN patients and biopsy controls compared with healthy controls. In addition, patients with IgAN had a significantly greater 24-h urinary protein excretion compared with biopsy controls.

**Table 2:** Demographic and clinical data from all subjects

Variables	Healthy controls	Biopsy controls	IgAN
	*n*=23	*n*=20	*n*=70
Age, years	36 (32–39)	39 (35–47)	36 (27–44)
Men, number (%)	12 (52)	12 (60)	40 (57)
MAP, mmHg	88 (86–91)	99 (97–106)^1^	100 (93–108)^1^
Serum creatinine, μmol/l	61 (54–72)	92 (78–109)^1^	86 (69–126)^1^
eGFR, ml/min/1.73m^2^	115 (112–118)	77 (65–95)^1^	83 (57–109)^1^
24-h urinary protein, g	0.04 (0.02–0.05)	0.09 (0.07–0.18)^1^	1.36 (0.65–2.78)^1,2^

Values are displayed as absolute numbers and percentages for gender and median (lower and upper quartiles) for all other variables. ^1^*P*<0.001 compared with healthy controls. ^2^*P*<0.001compared with biopsy controls.

### Renal *megalin* mRNA and miRNAs levels in IgAN

**Figure 1:**
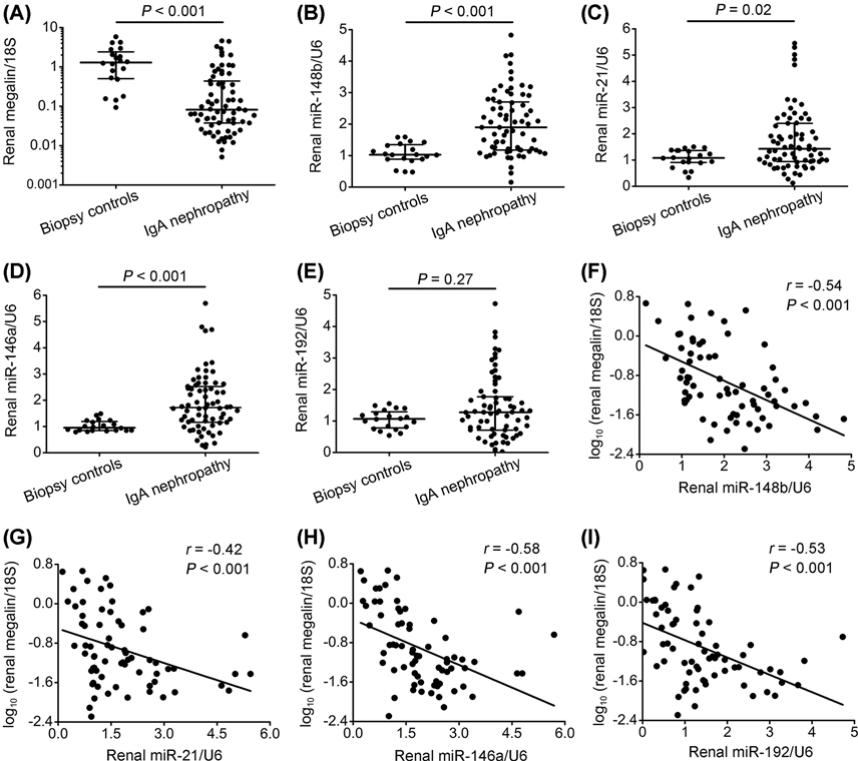
*Megalin* mRNA as well as miR-148b, miR-21, miR-146a, and miR-192 expression in kidney tissue from patients with IgAN (**A**) *Megalin* mRNA levels were decreased by 94% when compared with biopsy controls. (**B**–**E**) In contrast, levels of miR-148b, miR-21, and miR-146a were increased by 85%, 32%, and 80%, respectively, while a non-significant trend of increased miR-192 was observed in IgAN patients when compared with biopsy controls kidney tissue. (**F**–**I**) *Megalin* mRNA expression correlated inversely with levels of miR-148b, miR-21, miR-146a, and miR-192 in patients with IgAN. Renal *megalin* mRNA, miR-148b, miR-21, miR-146a, and miR-192 levels were detected by qPCR and normalized to 18S and U6, respectively. IgAN, *n*=70; biopsy controls, *n*=20. The horizontal lines (A–E) from top down represent 75th percentile, median, and 25th percentile; the vertical lines represent interquartile range. *Megalin* mRNA levels were logarithmically transformed to fit a normal distribution.

*Megalin* mRNA levels were significantly reduced in kidney biopsies from IgAN patients when compared with biopsy controls (Figure 1A), while the levels of miR-148b, miR-21, and miR-146a were significantly higher (Figure 1B–D). A trend toward increased renal miR-192 levels was also observed although not statistically significant (Figure 1E). We identified an inverse and significant correlation between the renal expression of *megalin* mRNA and all four miRNAs in patients with IgAN (Figure 1F–I); however, when adjusted for potential confounders using multiple linear regression analysis, only the correlation between renal miR-148b and *megalin* mRNA levels remained significant (Table 3). The results indicate that increased renal miR-148b levels are independently associated with decreased *megalin* mRNA levels.

**Table 3:** Multiple linear regression analysis of renal megalin expression with relevant miRNAs in IgAN

Variables	β	*P*-value	95% CI
Log_10_(renal miR-148b/U6)	−0.90	0.008	−1.56 to −0.24
Log_10_(renal miR-21/U6)	−0.05	0.88	−0.76 to 0.65
Log_10_(renal miR-146a/U6)	−0.79	0.09	−1.72 to 0.13
Log_10_(renal miR-192/U6)	−0.26	0.22	−0.67 to 0.16

Renal megalin mRNA (dependent variable) and levels of four different miRNAs (miR-148b, miR-21, miR-146a, and miR-192; independent variables) were measured by qPCR and normalized to 18S and U6, respectively. Data have been logarithmically transformed prior to multiple linear regression analysis using the Enter method. Abbreviation: 95% CI, 95% confidence interval.

**Figure 2:**
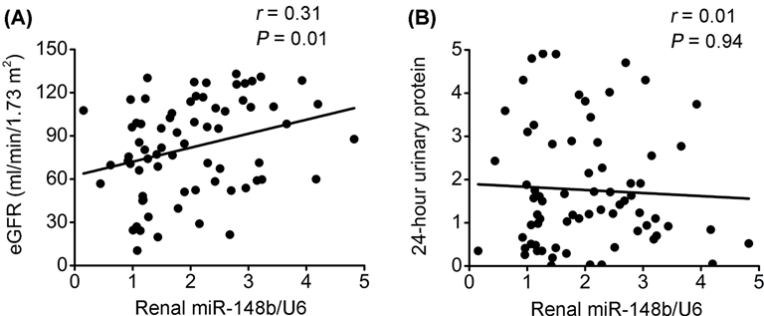
Correlations between renal miR-148b and eGFR or proteinuria in patients with IgAN. (**A**,**B**) Renal miR-148b levels correlated significantly with eGFR, but not with 24-h urinary protein excretion in patients with IgAN. Renal miR-148b levels were measured by qPCR and normalized to U6.

The renal miR-148b levels were correlated positively with eGFR, but not with 24-h urinary protein excretion (Figure 2). No correlation was observed between the levels of the other three renal miRNAs (miR-21, miR-146a, and miR-192) and eGFR or 24-h urinary protein excretion (data not shown).

### Renal megalin protein expression in patients with high or low levels of renal miR-148b

**Figure 3:**
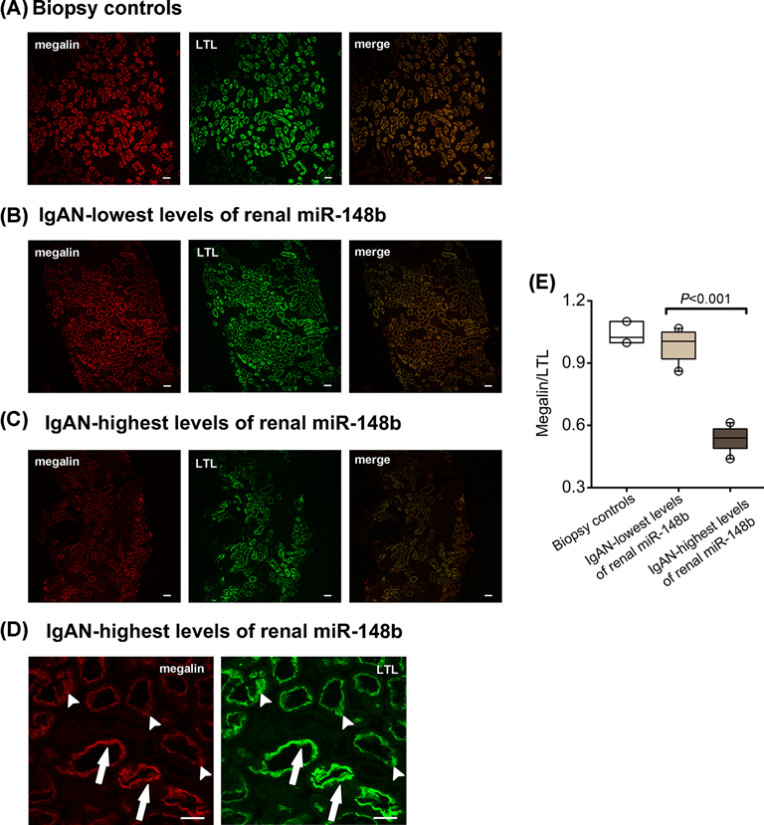
Renal megalin protein expression by immunofluorescence in biopsy controls and in IgAN patients with the highest or the lowest expression of renal miR-148b The images show double staining for megalin (red) and LTL (green) in kidney tissue from biopsy controls (**A**), IgAN patients with the lowest levels of renal miR-148b (**B**) or patients with the highest levels (**C**). Scale bars = 40 μm. (**D**) Higher magnification images from a patient belonging to the group with the highest levels of renal miR-148b. Some tubular profiles show intense labeling for both LTL and megalin (*arrows*), while others, that also show significant labeling for LTL, reveal much weaker labeling for megalin (*arrowheads*). Scale bars = 40 μm. (**E**) Quantitative analysis of megalin protein expression showing the ratios of megalin staining intensity to that of LTL in each section (*n*=3 for biopsy controls and *n*=10 for each of the two IgAN groups). Each box plot represents median, 25th and 75th percentiles. Whiskers represent 1.5-times interquartile range.

To further evaluate the possible correlation between megalin expression and renal miR-148b levels, we examined proximal tubule megalin protein expression by immunofluorescence in patients with the highest and the lowest levels of renal miR-148b, respectively. The eGFR, 24-h urinary protein and Oxford classification MEST scores were similar in these patients (Table 4). Megalin protein expression was normalized to the number of proximal tubule profiles assessed using LTL as a marker of the proximal tubule brush border (Figure 3A–D) [25]. In patients with the highest levels of renal miR-148b tubular profiles, which showed intense labeling for both LTL and megalin, were observed along with tubules revealing much weaker labeling for megalin but still intense labeling for LTL (Figure 3D). This suggests that the reduction in megalin labeling is not merely a reflection of decreased brush border integrity. Quantitation of the labeling revealed a significantly lower, relative intensity of megalin to LTL label intensity in patients with the highest levels of renal miR-148b when compared with the patients with the lowest levels (Figure 3E). This result is consistent with the notion that miR-148b may negatively regulate megalin expression in IgAN.

**Table 4:** Characteristics of IgAN patients with the highest and the lowest levels of renal miR-148b

Variables	IgAN- lowest levels of renal miR-148b, *n*=10	IgAN- highest levels of renal miR-148b, *n*=10	*P-*value
Age, years	40 ± 12	35 ± 9	0.28
Men, number (%)	6 (60)	5 (50)	1.00
MAP, mmHg	111 ± 10	97 ± 8	0.003
Serum creatinine, μmol/l	93 ± 27	92 ± 28	0.95
eGFR, ml/min/1.73 m^2^	83 ± 20	86 ± 29	0.81
24-h urinary protein, g	1.11 (0.39–2.72)	1.42 (0.68–3.01)	0.27
Renal megalin mRNA/18S	0.82 (0.13–1.33)	0.04 (0.02–0.11)	0.002
Oxford classification			
M0/M1, number	5/5	9/1	-
E0/E1, number	7/3	7/3	-
S0/S1, number	4/6	2/8	-
T0/T1/T2, number	7/1/2	7/2/1	-

Values are displayed as absolute numbers and percentages for gender and Oxford classification MEST scores, and mean ± S.D. or median (lower and upper quartiles) for all other variables. Renal *megalin* mRNA was measured by qPCR and normalized to 18S.

### Urinary excretion of miR-148b

miR-148b levels were significantly increased in urine from IgAN patients when compared with healthy controls (8.28 (6.5–9.8) versus 0.97 (0.78–1.08), *P*<0.001). However, urinary miR-148b levels did not correlate significantly with the renal levels in IgAN patients (data not shown).

### Effects of miR-148b, miR-21, miR-146a, and miR-192 on megalin expression in *vitro*

**Figure 4:**
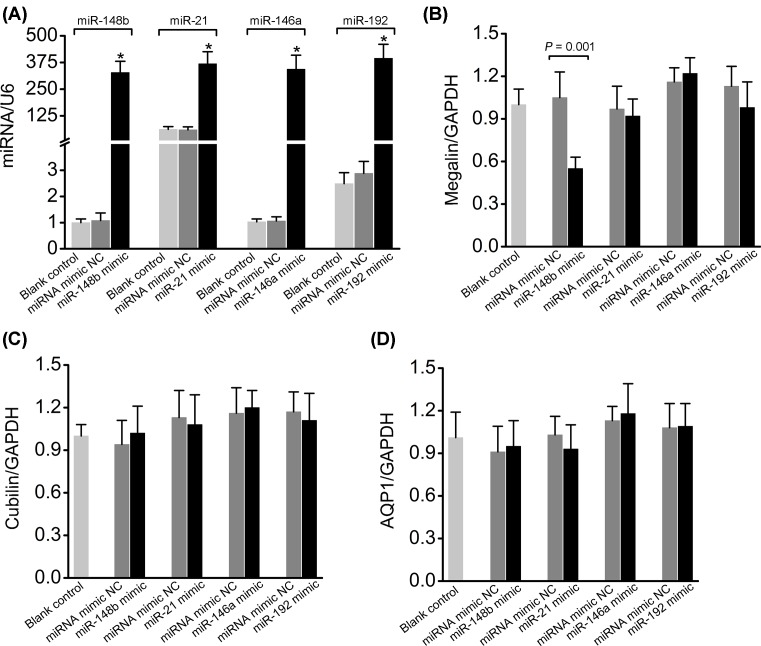
The effects of miR-148b, miR-21, miR-146a, and miR-192 mimics on the expression of megalin, cubilin, and AQP1 in LLC-PK1 cells (**A**) The expression of miR-148b, miR-21, miR-146a, and miR-192 was increased similarly in LLC-PK1 cells transfected with the corresponding miRNA mimics when compared with respective negative control (miRNA mimic NC). (**B**–**D**) Transfection with miR-148b mimic resulted in a significant decrease in endogenous *megalin* mRNA levels, but had no effect on cubilin or *AQP1* mRNA levels when compared with its negative control. In contrast, no significant effects on the levels of endogenous megalin, cubilin, or *AQP1* mRNA were observed following transfection with miR-21, miR-146a, or miR-192 mimic. The membrane proteins (megalin, cubilin, and AQP1) and miRNAs (miR-148b, miR-21, miR-146a, and miR-192) levels were measured by qPCR and normalized to GAPDH or U6. All values were presented as means ± S.D.; *n*=6 for each group. *P<0.001 when comparing corresponding miRNA mimic to the miRNA mimic NC.

To substantiate the independent association between renal miR-148b and megalin expression by functional analyses, we examined if miR-148b, miR-21, miR-146a, and miR-192 regulate the endogenous megalin expression *in vitro* by transfection of corresponding miRNA mimics into megalin expressing, proximal tubule LLC-PK1 cells [23]. Using this assay, we have previously identified miR-148b as a regulator of megalin expression [17]. Preliminary experiments revealed different transfection efficiency for the different miRNA mimics (data not shown). Thus, the transfection concentrations of the miRNA mimics and respective negative controls were adjusted to ensure comparable levels of the four miRNAs in the transfected LLC-PK1 cells (10, 7, 3, and 5 nM for the miR-148b, miR-21, miR-146a, and miR-192 mimics, respectively; Figure 4A). This should minimize the risk that differences in the overexpression levels by itself would lead to differential effects of the different miRNAs [26]. Transfection of LLC-PK1 cells with miR-148b mimic reduced *megalin* mRNA levels by approximately 50%, while miR-21, miR-146a, or miR-192 mimics did not significantly affect *megalin* mRNA expression (Figure 4B). No significant effect of transfection with any of the four miRNA mimics was observed on the mRNA levels of the membrane proteins cubilin and AQP1 when compared with their respective negative controls (Figure 4C,D). This suggests that cells are viable and that the effect of miR-148b on *megalin* mRNA expression is specific.

## Discussion

The present study shows a significant increase in the renal miR-148b, miR-21, miR-146a and urinary miR-148b levels associated with a reduced expression of *megalin* mRNA in kidney tissue from patients with IgAN. Multiple linear regression analysis revealed that renal miR-148b levels were independently and inversely correlated with *megalin* mRNA expression in IgAN. In addition, higher levels of renal miR-148b were associated with lower megalin protein expression by immunofluorescence. Furthermore, transfection with miR-148b, but not miR-21, miR-146a, and miR-192 specifically down-regulates *megalin* mRNA levels in renal proximal tubule cells *in vitro*, showing that amongst the tested miRNAs this effect on megalin is likely to be specific for miR-148b. These findings support the notion that miR-148b specifically and negatively regulates the expression of megalin in IgAN.

We have previously shown that miR-148b regulates megalin expression in proximal tubule cells *in vitro* and that increased miR-148b levels in experimental kidney injury induced by ureteral obstruction are associated with reduced megalin expression. Our present findings support this notion in human IgAN suggesting this to be a more general mechanism for regulating megalin expression in kidney diseases. Megalin dysfunction has been suggested in other acquired and common chronic kidney diseases including diabetic kidney disease [6,9,27–29], and may be regulated by multiple mechanisms [30,31].

Using the Oxford classification, an association between tubulointerstitial damage and progression of IgAN has been identified [32]. The severity of tubulointerstitial lesions is more closely related to disease progression than the glomerular lesions [33]. Tubulointerstitial damage is associated with increased urinary excretion of low molecular weight proteins [34]. Megalin plays a crucial role in reabsorption of filtered substances, including albumin, low-molecular-weight proteins, hormones, and vitamins. Recently, urinary megalin has been identified as a potential biomarker of tubular damage both in diabetic nephropathy and IgAN [9,11,27]. Moreover, Peters et al. [37] showed that urinary excretion of α1-microglobulin and β2-microglobulin, both being dependent on megalin for tubular reabsorption [35,36], correlated significantly with serum creatinine and predicted progression to ESRD in IgAN.

Recently, numerous studies have addressed the potential role of miRNAs in the pathogenesis and progression of IgAN [1,38,39]. Similar to our findings, an increased renal expression of miR-21, miR-146a, and miR-192, involved in renal fibrosis, have been reported in patients with IgAN [16,18,19,40]. In addition, their levels in kidney tissue were correlated with progression of IgAN. In this study only the renal levels of miR-148b were independently correlated with megalin expression without obvious difference in Oxford classification MEST scores between the patients with the highest levels of renal miR-148b and the lowest levels. While the low number of patients in each group greatly limits the interpretation of this, it may suggest that reduced megalin expression is not a result of the tubular damage only, but specifically related to the increased miR-148b. Other factors affecting megalin expression cannot be excluded, including the possible effects of other miRNAs not examined in the present study. MiR-146a was previously shown to target megalin in brain to increase cell apoptosis in Alzheimer’s disease [41]. Differences in cell signaling pathways may account for this apparent discrepancy between brain and kidney. Overexpression of miR-148b in peripheral blood mononuclear cells has also been shown to inhibit expression of the *C1GALT1* gene, increasing the circulating deglycosylated IgA1 which is regarded as the first and essential step in the development of IgAN [42]. Our findings provide evidence for an additional role of miR-148b on the pathophysiology of IgAN. Taken together, the modulation of miR-148b expression may be a relevant target for preventing or attenuating the development of IgAN by different pathways.

Urinary levels of miRNAs, such as miR-200a and miR-3613-3p, have been suggested to serve as biomarkers for diagnosis and monitoring of IgAN [43,44]. Yet, the source of urinary miRNAs still remains unclear. Several studies indicate that urinary miRNAs most likely originate from deciduous tubular epithelial cells, podocytes, or urinary erythrocytes [45,46]. In this study, urinary miR-148b levels did not correlate to renal miR-148b levels. Thus, while urinary miR-148b may at least in part originate from the renal tubule cells, there may be additional contributions from filtered miRNAs. Unfortunately, we did not have the opportunity to measure plasma miR-148b levels in the patients.

Our findings are limited by the single-center, cross-sectional design of the study including a limited number of Asian patients. Thus, prospective studies involving larger cohorts are needed further to validate the regulatory effect of miR-148b on proximal tubule megalin expression in IgAN in other populations and to evaluate the implications for disease progression. We have not directly established the clinical importance of miR-148b-mediated down-regulation of megalin in IgAN; however, given the essential role of megalin in proximal tubule physiology and likely role in progression of other types of kidney injury [10], we speculate that this specific regulation of megalin is likely to affect the pathophysiological process and therefore important. Further experimental work is required to confirm this.

In conclusion, our findings strongly support a role of miR-148b in the regulation of proximal tubule megalin expression in humans. Considering the essential role of megalin for the tubular uptake of filtered protein, hormones, and enzymes, this may have important implications for the renal metabolism and for the clearance of active substances within the renal tubular lumen. While still speculative, these findings suggest that restoring megalin may reduce the tubular dysfunction in the progression of IgAN. Prospective clinical studies and experimental work are needed to further explore this.

## Abbreviations

AQP1: aquaporin 1
eGFR: estimated glomerular filtration rate
ESRD: end-stage renal disease
IgAN: IgA nephropathy
LTL: lotus tetragonolobus lectin
MAP: mean arterial pressure
qPCR: quantitative PCR
